# Supplementary figures and images for: AL360181.1 promotes proliferation and invasion in colon cancer and is one of ten m6A-related lncRNAs that predict overall survival
Source: PeerJ. 2023 Nov 8;11:e16123. doi: 10.7717/peerj.16123 (PMC10638913; doi:10.7717/peerj.16123)

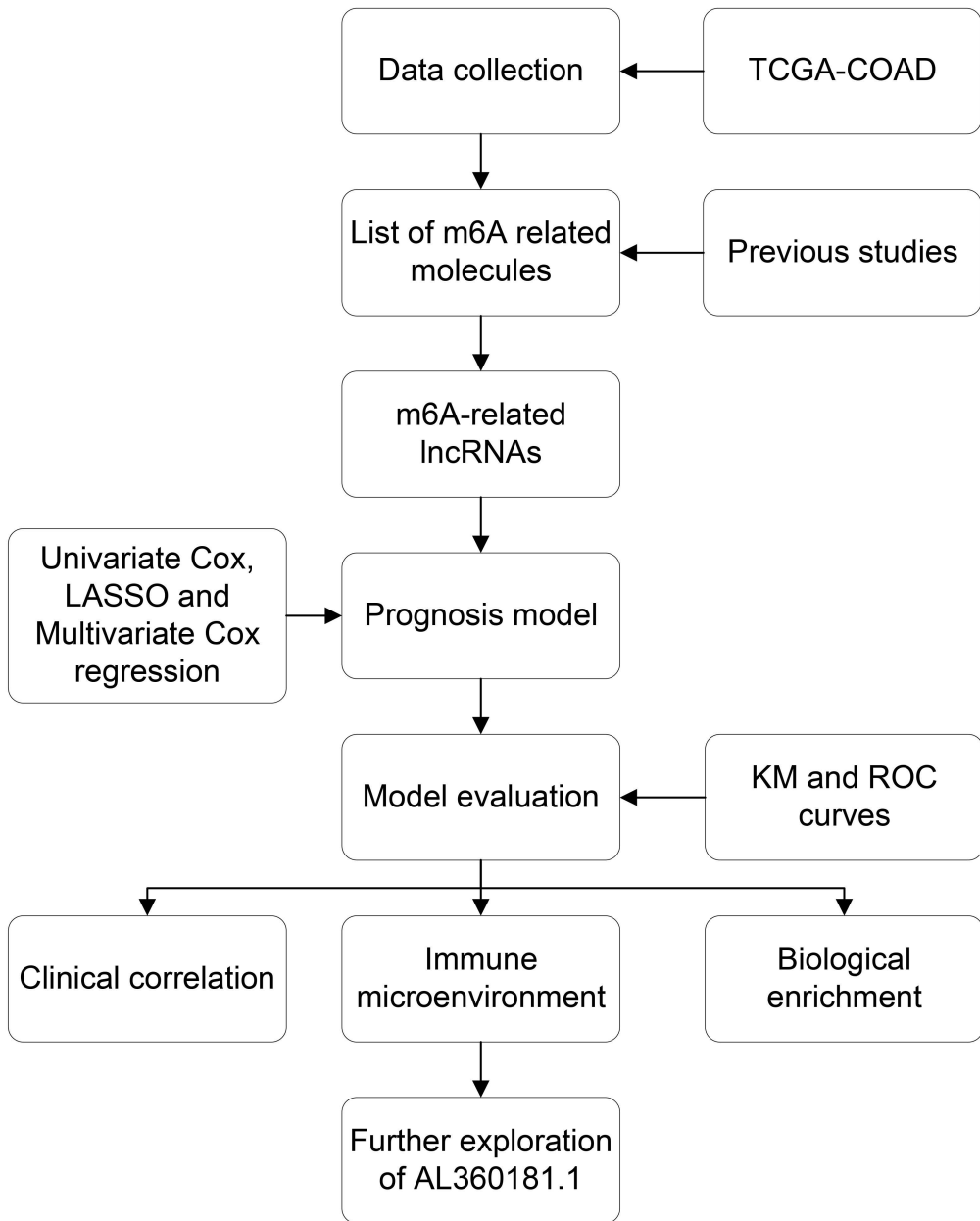

Supplement: Supplemental Information 1 [file peerj-11-16123-s001.pdf]

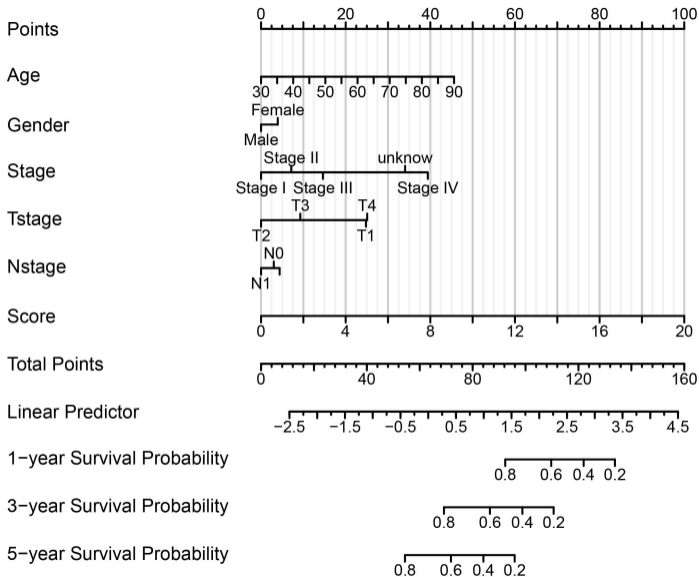

Supplement: Supplemental Information 2 [file peerj-11-16123-s002.pdf]
